# Twelve week liraglutide or sitagliptin does not affect hepatic fat in type 2 diabetes: a randomised placebo-controlled trial

**DOI:** 10.1007/s00125-016-4100-7

**Published:** 2016-09-15

**Authors:** Mark M. Smits, Lennart Tonneijck, Marcel H. A. Muskiet, Mark H. H. Kramer, Petra J. W. Pouwels, Indra C. Pieters-van den Bos, Trynke Hoekstra, Michaela Diamant, Daniël H. van Raalte, Djuna L. Cahen

**Affiliations:** 1 Diabetes Center, Department of Internal Medicine, VU University Medical Center, De Boelelaan 1117 (Room ZH 4A65), 1081 HV Amsterdam, the Netherlands; 2 Department of Physics and Medical Technology, VU University Medical Center and Neuroscience Campus Amsterdam, Amsterdam, the Netherlands; 3 Department of Radiology and Nuclear Medicine, VU University Medical Center, Amsterdam, the Netherlands; 4 Department of Health Sciences and the EMGO Institute for Health and Care Research, VU University Amsterdam, Amsterdam, the Netherlands; 5 Department of Epidemiology and Biostatistics, VU University Medical Center, Amsterdam, the Netherlands; 6 Department of Gastroenterology and Hepatology, Erasmus University Medical Center, Rotterdam, the Netherlands

**Keywords:** Dipeptidyl peptidase-4 inhibitor, Glucagon-like peptide-1 receptor agonist, Non-alcoholic fatty liver disease, Type 2 diabetes

## Abstract

**Aims/hypothesis:**

Glucagon-like peptide (GLP)-1-based therapies have been suggested to improve hepatic steatosis. We assessed the effects of the GLP-1 receptor agonist liraglutide and the dipeptidyl peptidase (DPP)-4 inhibitor sitagliptin on hepatic steatosis and fibrosis in patients with type 2 diabetes.

**Methods:**

In this 12 week, parallel, randomised, placebo-controlled trial, performed at the VU University Medical Center between July 2013 and August 2015, 52 overweight patients with type 2 diabetes treated with metformin and/or sulphonylurea agent ([mean ± SD] age 62.7 ± 6.9 years, HbA_1c_ 7.3 ± 0.7% or 56 ± 1 mmol/mol) were allocated to once daily liraglutide 1.8 mg (*n* = 17), sitagliptin 100 mg (*n* = 18) or matching placebos (*n* = 17) by computer generated numbers. Both participants and researchers were blinded to group assignment. Hepatic fat content was measured using proton magnetic resonance spectroscopy (^1^H-MRS). Hepatic fibrosis was estimated using three validated formulae.

**Results:**

One patient dropped out in the sitagliptin group owing to dizziness, but no serious adverse events occurred. At week 12, no between-group differences in hepatic steatosis were found. Liraglutide reduced steatosis by 10% (20.9 ± 3.4% to 18.8 ± 3.3%), sitagliptin reduced steatosis by 12.1% (23.9 ± 3.0% to 21.0 ± 2.7%) and placebo lessened it by 9.5% (18.7 ± 2.7% to 16.9 ± 2.7%). Neither drug affected hepatic fibrosis scores compared with placebo.

**Conclusions/interpretation:**

Twelve-week liraglutide or sitagliptin treatment does not reduce hepatic steatosis or fibrosis in type 2 diabetes.

***Trial registration*:**

ClinicalTrials.gov NCT01744236

***Funding*:**

Funded by the European Community’s Seventh Framework Programme (FP7/2007-2013) under grant agreement no. 282521 – the SAFEGUARD project.

**Electronic supplementary material:**

The online version of this article (doi:10.1007/s00125-016-4100-7) contains peer-reviewed but unedited supplementary material, which is available to authorised users.

## Introduction

Non-alcoholic fatty liver disease (NAFLD) is the most common chronic liver condition in the developed world, affecting over 30% of the population [1]. Its prevalence is particularly high among patients with type 2 diabetes mellitus (>70%), probably because of insulin resistance as a common denominator. NAFLD may progress to non-alcoholic steatohepatitis (NASH), cirrhosis and hepatocellular carcinoma, and is an independent risk factor for cardiovascular disease [1]. As such, it is associated with increased liver-related and all-cause mortality.

Lifestyle modifications, including dietary changes and weight loss, are the mainstay of NAFLD management, yet most patients with type 2 diabetes do not achieve or maintain their goals [1]. Moreover, although several pharmacological substances have been explored with promising results, including pioglitazone (reductions of up to approximately 50%), vitamin E and obeticholic acid [1], no drug is currently licensed for NAFLD. With an expected increase in prevalence of type 2 diabetes, NAFLD and consequent health risks, the search for effective and safe therapeutic strategies is ongoing.

Glucagon-like peptide (GLP)-1 receptor agonists and dipeptidyl peptidase (DPP)-4 inhibitors have been postulated as treatment options for NAFLD because of their positive effects on glycaemic control, body weight, insulin resistance, lipid metabolism and inflammation [2]. Encouraged by several experimental and open-label uncontrolled clinical studies [2], the current placebo-controlled randomised trial assessed the effects of the GLP-1 receptor agonist liraglutide and the DPP-4 inhibitor sitagliptin on spectroscopy-measured hepatic steatosis in patients with type 2 diabetes.

## Methods

Patients were evaluated in a 12-week, single-centre, randomised, placebo-controlled, double-blind, double-dummy, three-armed, parallel-group intervention trial. The study was approved by the local ethics review board, registered at ClinicalTrials.gov (NCT01744236) and conducted in accordance with the Declaration of Helsinki and the International Conference on Harmonization of Good Clinical Practice. All participants provided written informed consent before participation. This study was part of a larger trial performed between July 2013 and August 2015 at the VU University Medical Center, Amsterdam, the Netherlands, whose study objectives and protocol have previously been published [3]. Here, the effects of GLP-1-based therapies on the secondary endpoints of hepatic steatosis (measured using proton-magnetic resonance spectroscopy [^1^H-MRS]), hepatic fibrosis (assessed by fibrosis formulae) and hepatic function (from measurements of serum albumin and bilirubin) are reported.

### Study population

Patients with type 2 diabetes were eligible if they were aged between 35 and 75 years (and women were postmenopausal), had an HbA_1c_ level of 6.5–9.0% (48–75 mmol/mol), been treated with a stable dose of metformin and/or sulfonylurea derivatives for ≥3 months and had a BMI of 25–40 kg/m^2^. Relevant exclusion criteria were the use of GLP-1-based therapies or insulin, a history of hepatic or pancreatic disease, inability to undergo MRI scanning and alcohol intake >3 units/day [3].

### Intervention

After inclusion, a 4-week run-in period and baseline testing, patients were randomised by the trial pharmacist using computer-generated numbers (allocation 1:1:1, block size six) to receive the GLP-1 receptor agonist liraglutide 1.8 mg (Novo Nordisk A/S, Bagsvaerd, Denmark), the DPP-4 inhibitor sitagliptin 100 mg (Merck, Kenilworth, NJ, USA) or matching placebos, taken once daily in the evening. Endpoint measurements were repeated after 12 weeks of treatment.

### Endpoint measurements

Hepatic fat content was measured using proton ^1^H-MRS on a 1.5 T whole-body MRI scanner (Magnetom Avanto; Siemens Medical Solutions, Erlangen, Germany), with patients in the supine position and the body-array coil positioned at the upper abdominal region. Coronal and transverse structural T_2_-weighted images were used to localise a volume of interest (VOI). An 8 cm^3^ VOI (2 × 2 × 2 cm^3^) was selected at up to three locations in the liver (right superior, right inferior and left anterior), avoiding major blood vessels and bile ducts, and with sufficient distance from the liver edges. Using point-resolved spectroscopy sequences (echo time 30 ms, retention time 2000 ms, during free breathing, no gating), single voxel spectra were recorded. Eight acquisitions were obtained per VOI, and stored separately. User-independent spectral quantification was performed with LCModel (version 6.1; available from http://s-provencher.com).

Fat content was expressed as the percentage of the area under the methyl (0.9 ppm) and methylene (1.3 ppm) peaks, relative to the area under the water (4.65 ppm) peak. For each VOI, individual acquisitions were reviewed in order to discard occasional poor-quality spectra (e.g. due to motion artefacts). Individual spectra were combined to obtain the fat percentage for each VOI. The mean fat content of the available VOIs was used. Variation was assessed in 11 patients who underwent an additional baseline ^1^H-MRS session within 3 weeks as part of a different study [3]. The within-VOI variation was 6.7%, the between-VOI variation 12.2% and the variation in liver fat (combination of three VOIs; day-to-day variance) 11.7%.

Hepatic fibrosis was estimated at baseline and 12 weeks using validated formulae: the NAFLD fibrosis score (NFS), Fibrosis-4 (FIB-4) score and aspartate aminotransferase to platelet ratio index (APRI) [4]. Fasting blood samples were drawn for measurement of glucose (gluco quant-hexokinase method), HbA_1c_ (HPLC), insulin (immunometric method), (cholestatic) liver enzymes (enzymatic assessment), albumin, total and conjugated bilirubin (colorimetric measurement), thrombocyte count (laser light scattering) and liver fatty acid binding protein (L-FABP; using sandwich ELISA).

### Sample size, data management and statistical analysis

Sample size calculations have previously been published [3]. With an expected reduction in the percentage of steatosis of approximately 50% [5], a total of 13 patients per treatment arm were needed (parallel-group design, α = 0.05, power [1 − β] 80%). To test treatment effects vs placebo, multivariable regression analyses were performed on the per-protocol population, using SPSS 22 (IBM SPSS, Chicago, IL, USA). Treatment with liraglutide or sitagliptin was added as a dummy variable. To correct for baseline differences, pretreatment values were included in the model. Moreover, proportions of patients with any improvement in steatosis in each treatment group were compared using the *χ* ^2^ test. Analyses were repeated in the subgroup with NAFLD (>5.56% steatosis) at baseline. A two-sided *p* ≤ 0.05 was considered to be statistically significant.

## Results

**Fig. 1:**
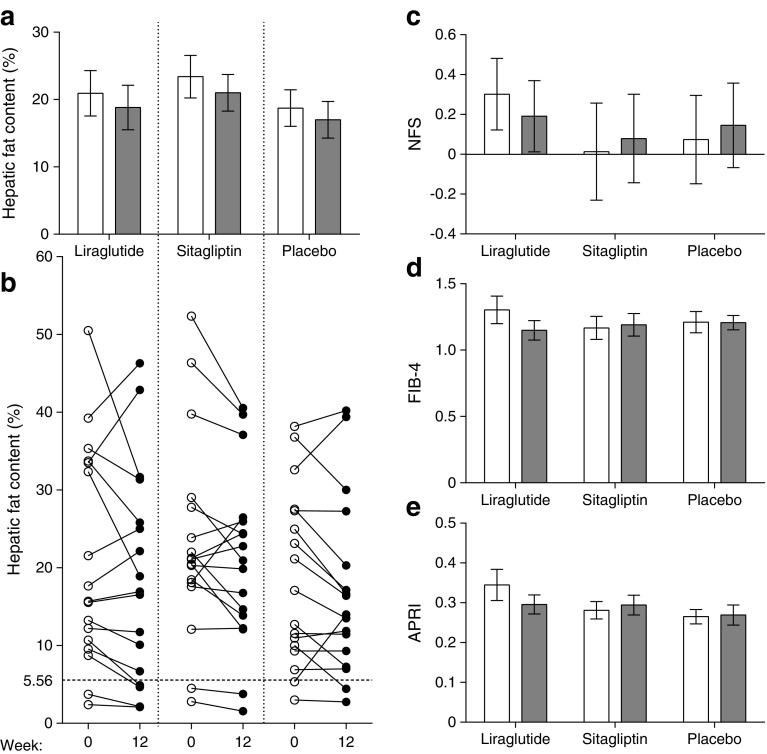
Effects of treatment on ^1^H-MRS-measured hepatic fat content and calculated fibrosis. Effects of liraglutide, sitagliptin or placebo on hepatic endpoints. White bars, measurements at baseline; grey bars, measurements at 12 weeks. (**a**, **b**) Hepatic fat content as measured using ^1^H-MRS, with individual effects shown in (**b**). Markers of hepatic fibrosis: (**c**) NFS; (**d**) FIB-4; (**e**) APRI. Data are mean ± SEM. None of the effects was statistically significant (*p* < 0.05)

Of the 52 patients who were randomised, one patient in the sitagliptin group withdrew from the study because of adverse effects (dizziness and daytime urinary frequency). Fifty-one patients completed the 12 week study (17 per treatment arm) (see electronic supplementary material [ESM] Fig. 1). The baseline characteristics were similar between the groups (Table 1). Liraglutide and sitagliptin reduced fasting glucose (−1.6 ± 0.5 mmol/l and −1.8 ± 0.5 mmol/l, respectively; both *p* < 0.001) and HbA_1c_ (−1.3 ± 0.2% [−14 ± 2 mmol/mol] and −0.9 ± 0.2% [−10 ± 2 mmol/mol], respectively; both *p* < 0.001), compared with placebo. Liraglutide tended to reduce the participant’s weight (−1.9 ± 1.0 kg; *p* = 0.06), whereas sitagliptin was weight-neutral (−0.6 ± 1.0 kg; *p* = 0.56).

**Table 1:** Baseline characteristics and treatment-induced effects

	Liraglutide (*n* = 17)			Sitagliptin (*n* = 17)			Placebo (*n* = 17)	
Parameter	Baseline	Follow-up	Placebo-corrected mean difference	Baseline	Follow-up	Placebo-corrected mean difference	Baseline	Follow-up
Participants’ characteristics								
Age (years)	60.8 ± 1.8			61.5 ± 1.7			65.8 ± 1.4	
Male sex (*n* (%))	12 (70.6)			14 (82.4)			13 (76.5)	
T2DM duration (years)	7.9 ± 1.2			8.5 ± 1.5			8.2 ± 1.2	
Metformin (*n* (%))	17 (100%)			16 (94.1%)			15 (88.2%)	
Sulfonylurea (*n* (%))	6 (35.3%)			9 (52.9%)			8 (47.1%)	
Hepatic fat content								
^1^H-MRS (%)	20.9 ± 3.4	18.8 ± 3.3	−0.04 ± 1.86 (*p* = 0.98)	23.9 ± 3.0	21.0 ± 2.7	0.05 ± 1.87 (*p* = 0.98)	18.7 ± 2.7	16.9 ± 2.7
Hepatic fibrosis formulae								
NFS	0.24 ± 0.18	0.10 ± 0.19	−0.25 ± 0.14 (*p* = 0.08)	0.05 ± 0.27	0.04 ± 0.24	−0.07 ± 0.13 (*p* = 0.61)	0.07 ± 0.22	0.15 ± 0.21
FIB-4	1.3 ± 0.1	1.1 ± 0.1	−0.13 ± 0.08 (*p* = 0.12)	1.2 ± 0.1	1.2 ± 0.1	−0.00 ± 0.08 (*p* = 0.99)	1.2 ± 0.1	1.2 ± 0.1
APRI	0.3 ± 0.03	0.3 ± 0.02	−0.01 ± 0.02 (*p* = 0.79)	0.3 ± 0.02	0.3 ± 0.02	0.03 ± 0.02 (*p* = 0.19)	0.3 ± 0.02	0.3 ± 0.03
Anthropometrics								
Weight (kg)	103.2 ± 3.2	101.0 ± 3.1	−1.89 ± 0.99 (*p* = 0.06)	98.5 ± 4.4	97.7 ± 4.5	−0.57 ± 0.97 (*p* = 0.56)	95.8 ± 2.4	95.7 ± 2.5
BMI (kg/m^2^)	32.8 ± 1.0	32.2 ± 1.0	−0.51 ± 0.33 (*p* = 0.13)	31.4 ± 1.1	31.1 ± 1.1	−0.20 ± 0.35 (*p* = 0.54)	30.6 ± 0.7	30.5 ± 0.8
Laboratory tests								
Glucose (mmol/l)	8.3 ± 0.3	7.3 ± 0.4	−1.61 ± 0.47 (*p* = 0.001)*	7.9 ± 0.2	7.0 ± 0.2	−1.82 ± 0.49 (*p* < 0.001)*	8.9 ± .5	9.4 ± 0.5
Insulin (pmol/l)	78.8 ± 9.7	113.7 ± 16.2	52.6 ± 11.4 (*p* < 0.001)*	103.6 ± 15.3	113.3 ± 18.4	28.0 ± 11.2 (*p* = 0.016)*	106.4 ± 17.4	87.9 ± 13.4
HbA_1c_ (%)	7.4 ± 0.2	6.7 ± 0.2	−1.26 ± 0.21 (*p* < 0.001)*	7.1 ± 0.1	6.7 ± 0.1	−0.91 ± 0.22 (*p* < 0.001)*	7.5 ± 0.2	8.0 ± 0.3
HbA_1c_ (mmol/mol)	57 ± 2	49 ± 2	−14 ± 2 (*p* < 0.001)*	54 ± 1	49 ± 1	−10 ± 2 (*p* < 0.001)*	58 ± 2	64 ± 4
AST (U/l)	24.2 ± 1.9	22.4 ± 1.4	0.09 ± 1.82 (*p* = 0.96)	22.8 ± 1.4	23.5 ± 2.1	2.16 ± 1.79 (*p* = 0.23)	22.2 ± 1.8	21.6 ± 1.7
ALT (U/l)	28.9 ± 2.9	29.4 ± 3.2	3.38 ± 2.59 (*p* = 0.19)	28.9 ± 3.1	30.0 ± 3.7	4.07 ± 2.59 (*p* = 0.12)	32.0 ± 5.2	28.5 ± 3.9
γGT (U/l)	68.2 ± 3.8	67.6 ± 2.9	1.63 ± 2.50 (*p* = 0.52)	67.1 ± 4.5	64.1 ± 4.7	−0.92 ± 2.49 (*p* = 0.71)	62.6 ± 2.9	61.4 ± 2.5
ALP (U/l)	31.4 ± 3.0	28.2 ± 2.8	−2.77 ± 3.51 (*p* = 0.43)	45.0 ± 5.9	42.2 ± 5.4	−0.05 ± 3.47 (*p* = 0.99)	43.2 ± 10.3	40.8 ± 9.1
Albumin (g/l)	37.0 ± 0.4	35.8 ± 0.5	−1.59 ± 0.56 (*p* = 0.007)*	36.4 ± 0.6	35.8 ± 0.5	−1.22 ± 0.56 (*p* = 0.036)*	37.0 ± 0.6	37.4 ± 0.5
Total bilirubin (μmol/l)	10.4 ± 1.2	7.6 ± 0.9	−1.02 ± 0.88 (*p* = 0.25)	9.3 ± 1.4	8.0 ± 1.3	0.14 ± 0.86 (*p* = 0.87)	7.4 ± 0.6	6.6 ± 0.5
Conjugated bilirubin (μmol/l)	4.0 ± 0.4	3.2 ± 0.3	−0.48 ± 0.33 (*p* = 0.15)	3.7 ± 0.4	3.3 ± 0.4	−0.07 ± 0.32 (*p* = 0.83)	3.0 ± 0.2	2.9 ± 0.2
L-FABP (ng/ml)	9.4 ± 1.2	8.3 ± 1.4	1.33 ± 1.57 (*p* = 0.40)	10.1 ± 2.5	8.9 ± 1.2	1.82 ± 1.58 (*p* = 0.26)	8.4 ± 1.1	6.4 ± 0.8

Data are presented as mean ± SEM for continuous data

*p* values represent differences between treatment and placebo, corrected for baseline values. *Statistically significant at *p* < 0.05

FLI, fatty liver index; T2DM, type 2 diabetes

Twelve-week treatment with liraglutide or sitagliptin did not affect hepatic steatosis (−10% and −12.1% from baseline, respectively), no different from placebo (−9.5%; *p* = 0.98 for both) (Table 1, Fig. 1). In addition, the proportion of patients with any reduction in hepatic fat content was similar in the three groups: liraglutide 60.0%, sitagliptin 62.5%, and placebo 68.8%.

Neither liraglutide nor sitagliptin affected NFS, FIB-4 or APRI compared with placebo (all *p* > 0.05) (Table 1). No treatment-induced differences occurred in plasma aspartate aminotransferase (AST), alanine aminotransferase (ALT), gamma glutamyl transferase (γGT), alkaline phosphatase (ALP) or L-FABP concentrations (Table 1). Compared with placebo, liraglutide and sitagliptin reduced plasma albumin levels (−1.4 ± 0.6 g/l, *p* = 0.03 and −1.7 ± 0.6 g/l, *p* = 0.01, respectively), without affecting total or conjugated bilirubin (*p* > 0.05 for both).

As shown in Fig. 1, baseline hepatic fat content (Fig. 1a) did not determine treatment effects . Moreover, a subanalysis of patients with NAFLD at baseline (liraglutide *n* = 15, sitagliptin *n* = 16, placebo *n* = 15) did not yield different results (data not shown).

## Discussion

In the current study, we did not observe a beneficial effect of 12 week liraglutide or sitagliptin treatment on hepatic steatosis or fibrosis. Although high-quality human studies were lacking when the present study was designed, several randomised controlled trials have in the meantime been performed and published. In line with our findings, Tang et al demonstrated no effect of 12 week treatment with liraglutide on MRI-measured liver fat [6]. Moreover, 24 week treatment with sitagliptin did not affect liver fat in patients with NAFLD when compared with placebo [7]. In contrast, a 48 week double-blind, placebo-controlled trial with liraglutide demonstrated an improvement in steatosis and a histological resolution of NASH in patients with and without type 2 diabetes [8]. Also, 26 week treatment with exenatide reduced hepatic fat by 24% [9]. A 6 month, double-blind, placebo-controlled, randomised controlled trial reported a 27% reduction in hepatic fat with vildagliptin that was unrelated to changes in body weight [10]. Finally, an open-label trial comparing sitagliptin with glimepiride found no change in intrahepatic fat content after a 12 week treatment, but reported a 15% decrease after 24 weeks [11]. These contradictory outcomes may result from differences in treatment duration, study population and the method used to assess hepatic fat.

Albeit only as statistical trend, liraglutide decreased body weight within the expected range [12]. In recent studies with liraglutide [8, 13], weight loss reached a nadir after 24 weeks, indicating that further weight loss could have been observed with a longer duration of the current trial. Since weight loss per se reduces hepatic steatosis [14], a longer trial might have yielded different results.

Unexpectedly, we observed a modest decrease in plasma albumin levels in the liraglutide and sitagliptin groups, for which we cannot provide an explanation. Inhibition of hepatic synthetic function seems unlikely, as bilirubin levels remained unchanged and an increased risk of bleeding due to GLP-1 based therapies has never been reported. In addition, other potential causes, such as inflammation or renal loss of albumin, were not observed.

The limitations of the current study were untriggered magnetic resonance spectroscopy measurements and manual VOI positioning. To diminish the effect of small variations before and after intervention, we averaged over three positions in the liver. Proton-density fat fraction MRI is an alternative and widely available option [15]. Although the sample size was small, it is unlikely that a larger trial would have yielded different results given the minimal between-group differences.

In conclusion, 12 week treatment with liraglutide or sitagliptin did not improve hepatic steatosis or fibrosis in overweight patients with type 2 diabetes. Further, longer term studies are needed to assess the potential of these agents as treatment strategy for NAFLD.

## Electronic supplementary material

Below is the link to the electronic supplementary material.

ESM Fig. 1 (PDF 250 kb)

## Abbreviations

^1^H-MRS: Hydrogen magnetic resonance spectroscopy
γGT: Gamma glutamyl transferase
ALP: Alkaline phosphatase
ALT: Alanine aminotransferase
APRI: Aspartate aminotransferase to platelet ratio index
AST: Aspartate aminotransferase
DPP-4: Dipeptidyl peptidase-4
FIB-4: Fibrosis-4
GLP-1: Glucagon-like peptide-1
L-FABP: Liver fatty acid binding protein
NAFLD: Non-alcoholic fatty liver disease
NASH: Non-alcoholic steatohepatitis
NFS: NAFLD fibrosis score
VOI: Volume of interest
