# Supplementary figures and images for: Twelve week liraglutide or sitagliptin does not affect hepatic fat in type 2 diabetes: a randomised placebo-controlled trial
Source: Diabetologia. 2016 Sep 15;59(12):2588–93. doi: 10.1007/s00125-016-4100-7 (PMC6518065; doi:10.1007/s00125-016-4100-7)

**ESM Figure 1: flow chart**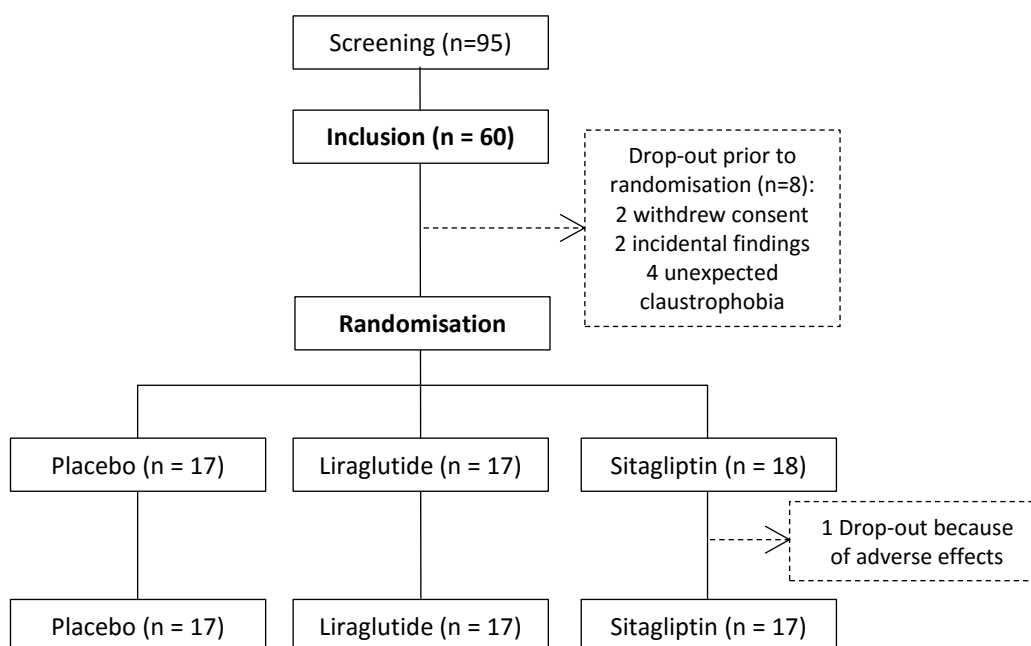

Supplement: Supplementary file 1 — (PDF 250 kb) [file 125_2016_4100_MOESM1_ESM.pdf]
